# The role of learning in farmer-led innovation

**DOI:** 10.1016/j.agsy.2021.103356

**Published:** 2022-03

**Authors:** Jonathan Ensor, Annemarieke de Bruin

**Affiliations:** Stockholm Environment Institute, Department of Environment and Geography, University of York, UK

## Abstract

**CONTEXT:**

Farmer-led innovation brings farmers together with other stakeholders in a collaborative endeavour that recognises multiple forms of expertise. Critical engagement with mainstream models of agricultural science and technology (AST) development has drawn attention to the isolation of farmers as technology adopters within a compartmentalised model of AST development and dissemination. Academic, government and non-governmental actors and organisations are increasingly supporting facilitated processes in which farmers, scientists and engineers develop new knowledge, learning together about the nature of the problems being faced and the potential of different solution pathways.

**OBJECTIVE:**

Despite the centrality of learning to farmer-led innovation, its role has yet to be systematically explored. In response, this paper looks to understand the forms of learning and their contribution to farmer-led innovation during a three-year action-research project involving two groups of farmers from northern England and the Scottish Borders in the UK.

**METHODS:**

A researcher-facilitator convened a structured process of twenty meetings that together created opportunities for interaction, deliberation and re-framing of problems and solutions among groups of farmers, a university-based engineer, and wider stakeholders. Multiple qualitative methods were used to build understanding of the different farming contexts and to explore the issues the farmers wanted to work on. Meeting transcripts and fieldnotes were subject to thematic analysis, informed by the analytical framework of cognitive, normative and relational learning derived from the social learning literature.

**RESULTS AND CONCLUSIONS:**

Cognitive, normative and relational learning were found to be mutually interdependent and equally significant, building iteratively rather than linearly: the farmers and engineer assessed new information and reappraised existing situations; they did so informed by and informing a shift in understanding of their goals for new technology; and in so doing they relied on and developed the trust and confidence needed to acknowledge or challenge each other's perspectives. By orientating the group engagement process around the space to explore and challenge histories and contexts of AST, and by drawing on social learning principles to facilitate interaction between the different expertise of farmers and between farmers and engineers, learning emerged that interleaved technology co-design with incremental refinement of the shared norms and values embedded in the process itself.

**SIGNIFICANCE:**

A focus on learning helps deepen understanding of key mechanisms and processes that define and deliver innovation, and the findings suggest that priorities for farmer-led innovation process design should focus on modalities that open up spaces to negotiate both the purpose and products of innovation.

## Highlights

• Farmer-led innovation is increasingly supported, relying on farmers and stakeholders learning together.
• The forms of learning and their contribution to innovation are overlooked in research and practice.
• Cognitive, normative and relational learning were mutually interdependent and equally significant in two farmer groups.
• Learning was iterative, interleaving technology co-design with refinement of shared norms and values.
• Opening spaces that recognise power and context allows negotiation of purpose and products of innovation processes.

## Graphical abstract

**Figure:**
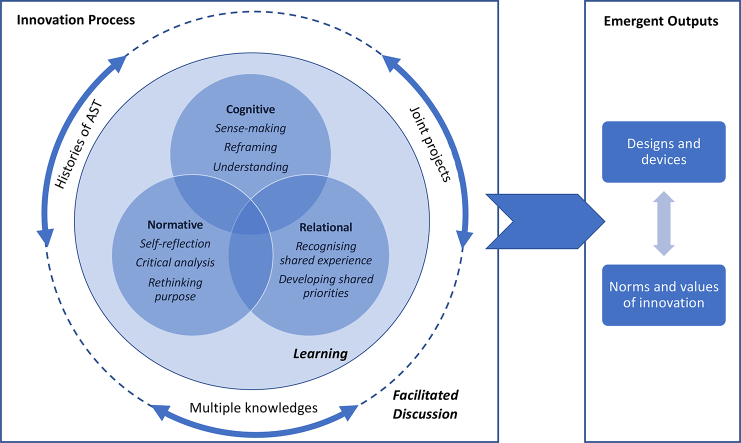

## 1 Introduction

Farmer-led innovation, in which farmers play a central role in the generation of new knowledge, technologies and ways of working, is increasingly recognised as necessary to secure the social, economic and environmental sustainability of farming. The model of agricultural science and technology (AST) as a centralized activity, undertaken by experts and diffused to farmers, has been challenged by collaborative research and innovation approaches in which farmers, scientists and engineers can learn together about the nature of the problems being faced and the potential of different solution pathways (Berthet et al., 2018; Caron et al., 2014; Macmillan and Benton, 2014). Yet despite the centrality of learning to farmer-led innovation, the literature has yet to unpack what is learnt during these processes, limiting understanding of process and impact ( de Bruin and Ensor, 2018; Ensor and Harvey, 2015). While tangible changes to the objects and practices used on farm are important, these changes are themselves rooted in what was learned: was new knowledge generated in relation to a particular problem? Was the problem understood in new ways? Were new relationships forged in addressing the problem? This paper addresses these questions, tracing forms of learning and their contribution to farmer-led innovation during a three-year action-research project involving two groups of farmers engaged in technology co-design in northern England and the Scottish Borders in the UK.

Mainstream approaches to AST have increased labour productivity, lowered food prices and, until recently, improved yields. Yet increasingly there is recognition that farmers are struggling to meet diverse challenges, including those posed by climate change, environmental degradation, escalating livelihood vulnerability and a rapidly shifting regulatory and market environment. Critical engagement with the trajectory of agricultural development has intensified, drawing attention in particular to the isolation of farmers as technology adopters within a compartmentalised model of AST development and dissemination (van Dijk et al., 2017; Jiggins et al., 2016; Pelletier et al., 2016; Schut et al., 2014; Tambo, 2018). Mirroring patterns of concentration throughout the supply chain, the diversity of knowledge and information brought to AST has contracted as the power of commercial interests has increased in contemporary food systems and as AST investment has focused on increasingly small numbers of research centres and towards high-technology instruments and techniques (Macmillan and Benton, 2014; Rotz and Fraser, 2015). For farmers, the effect is felt in the provision of generalised and standardised packages of technology and practice options that are designed far from the site of application and may poorly reflect their contexts and interests (Macmillan and Benton, 2014; van Dijk et al., 2017; Berthet et al., 2018).

Interest in farmer-led innovation has grown in response to these trends. A growing literature now documents diverse examples (van Dijk et al., 2017; Lowe et al., 2019; Macmillan and Benton, 2014; Naouri et al., 2020; Tambo and Wünscher, 2017; Waters-Bayer et al., 2015), reflecting widespread interest among governmental stakeholders including the EU (European Commission, 2016) and the UK's Department for Environment Food and Rural Affairs (DEFRA, 2020), and the promotion of farmer-led approaches by non-governmental actors (Innovative Farmers, 2020). In some settings, farmer-led innovation is used to refer to changes that emerge from farmers without explicit engagement with other actors (e.g. Tambo and Wünscher, 2017). Here, our interest is in farmer-led innovations (that is, new ways of doing things) to emerge from what Waters-Bayer et al. (2015, p.2) refer to as farmer-led research (that is, “a process in which farmers together with other support agents (for example, formal researchers, extensionists) investigate possible ways to improve the livelihoods of local people in the realm of agriculture and natural resource management”). Farmer-led innovation is, thus, “the process of developing locally new and better ways of doing things” (Waters-Bayer et al., 2015, p.2), where collaboration occurs but emphasis is placed on the role of farmers in driving knowledge production (Woodhouse et al., 2016; de Bont et al., 2019). While we recognise that innovations may be exclusively social, institutional and/or technical in character, the cases reported here centred on technology design and development that is anchored in and influences the social and institutional setting.

This paper responds to calls for “more open, decentralized, contextualized and participatory approaches to design and technology development, and more broadly innovation in agricultural systems” (Berthet et al., 2018 p.111). While recognising that agricultural innovations are ultimately co-determined by interactions between the actors, policies and institutions within wider systems (Klerkx et al., 2012; Leeuwis, 2004; Roling, 2009), the analytical focus in the following is on small networks of farmers and the relationships that they secure through a facilitated farmer-led innovation process. Each group worked with a dedicated engineer, and focused on the potential for improvements in their farm livelihoods and welfare through novel applications of AST. These groups were not conceptualised as playing a specialised role within the larger agriculture innovation system; rather, the shared aim of the group members and researchers (the latter being the authors of this paper) was to explore the significance and potential of a farmer-led approach to innovation, with particular focus on placing farmers at the centre of defining problems and solutions (Klerkx et al., 2012). The emphasis of the facilitation methods was on co-design as a mechanism towards “developing locally new and better ways of doing things” (Waters-Bayer et al., 2015 p.2), where co-design implies the “collective exploration of solutions to a common problem [that] seeks to build and maintain a shared conception of the design problem to allow collaboration” (Berthet et al., 2018, p.112). While systemic and institutional change were beyond the scope of the facilitated innovation projects, emphasis was placed on building the legitimacy of farmer experiences and knowledge among the engineers and research scientists that were brought into the process, against the backdrop of contextual shifts in the policy and funding environment towards farmer-led innovation. The wider goal of the innovation process was to provide evidence in support of greater farmer involvement in AST; this goal was embraced by the farmers involved in the two case studies, with many subsequently volunteering to participate in meetings with policy makers to discuss their experiences and insights. More specifically, the focus offered the opportunity to develop insights into the forms of learning that can take place within, and go towards defining, farmer-led innovation. As such it contributes to a growing evidence base on the mechanisms and processes at play in agricultural innovation systems (Klerkx et al., 2012).

The next section places farmer-led innovation in the context of literature on social learning. This literature understands learning to result from “collaborative processes that allow a shared sense of meaning to be arrived at by the community” (Ensor and Harvey, 2015, p. 510). It recognises that knowledge is situated, emerging within and structured by the interaction between actors. In the two case studies that are the focus of the current paper, this interaction was between a researcher-facilitator, farmers, an engineer who also operated as a link to scientists working on different aspects of AST, and wider stakeholders who were invited to join the meetings when the groups felt that different forms of expertise were required. The design of the process was conceptualised as a “social learning approach”, with facilitation intended to create the conditions in which social learning may occur (Ensor and Harvey, 2015, p. 510). The literature review concludes by setting out a framework for analysing learning, drawn from the social learning literature, which is subsequently applied to structure the results and discussion of the farmer-led innovation process in 3, 4.

## 2 Farmer-led innovation and social learning

It is now increasingly recognised that new methods are required to ensure the knowledge, motivations and interests of farmers and other stakeholders are integrated into innovation if desired impacts and shared goals are to be achieved (Berthet et al., 2018; Roling, 2009; Klerkx et al., 2012; Macmillan and Benton, 2014; Jiggins and Visser, 2016). This builds on a substantial history of critical engagement within and beyond AST that recognises the complexity and uncertainty inherent to environmental challenges (Funtowicz and Ravetz, 1994). Experts, policy makers and wider stakeholders have become engaged in problem solving as part of an “extended peer community” (see: Turnpenny et al., 2009, Turnpenny et al., 2010) to help tackle complexity and in recognition of the presence of diverse value systems (Jasanoff, 2003; Nowotny, 2003). A congruent literature has emerged in relation to climate change (Collins and Ison, 2009; Pasquier et al., 2020), water catchment management (Ison et al., 2007; Lumosi et al., 2019), and sustainability science (Lang et al., 2012). In each case, there is a recognition that multiple stakeholders, often with diverse perspectives, are implicated in defining and resolving problems. Tacit or experiential knowledge is valued alongside that produced by science (Kolb, 1984); innovation in policy or practice occurs through the interaction of formal/explicit knowledge with knowledge that is developed through the practices of particular groups of skilled individuals (Tsoukas and Vladimirou, 2001; Berthet et al., 2018; Colvin et al., 2014). While not suggesting the presence of rigid boundaries between the knowledge of scientists and other stakeholders (Agrawal, 1995), these multi-stakeholder innovation processes acknowledge the expertise of people that live and work in the problem settings that science would otherwise objectify (Lowe et al., 2019).

This trend is increasingly evident in literature concerned with the social and environmental sustainability of food and agriculture (Jiggins et al., 2016). The emergence of theoretical framings for innovation as *agricultural knowledge and innovation systems* in the 1990s and *agricultural innovation systems* in the 2000s charts a shift towards collaborative and co-development approaches. In these framings, joint knowledge production and shared learning take centre stage (Klerkx et al., 2012), albeit within wider debates around the appropriate scale of innovation processes and the necessity of systemic and institutional change (Jiggins et al., 2016; Jiggins and Visser, 2016). Sustaining or securing gains in production while meeting the challenges of economic, social and environmental sustainability requires “innovating in context” (Macmillan and Benton, 2014, p. 25), with farmers active in co-producing knowledge alongside research and technology specialists (e.g., Caron et al., 2014). This literature reflects the growing significance attached to rural, place-based “vernacular expertise” and the diverse ecologies, knowledge and expertise found in farm environments (Lowe et al., 2019, p. 28).

The implication is that the rules, norms and methods of innovation need to shift so that diverse forms of expertise can be recognised, building new relationships, expanding problem framings and opening space for new solutions to problems of food production and the management of natural resources (Jiggins et al., 2016). In this context, interest in farmer-led innovation has increased in the research, policy and NGO communities. As a process in which farmers work together with others (Waters-Bayer et al., 2015), farmer-led innovation implies that multiple forms of expertise are recognised and knowledge is combined and co-developed between differently situated partners (Lowe et al., 2019; Naouri et al., 2020; Stringer and Reed, 2007). However, within these collaborations farmers are recognised as both the users *and* producers of innovations, in a process that is “democratised” relative to mainstream AST (Naouri et al., 2020 p.2; Schut et al., 2014). This framing is significant as it emphasises the centrality of farmer agency (Veldwisch et al., 2019): for innovation to be meaningfully ‘farmer-led’ it must be farmers who drive the processes of problem definition and knowledge generation (Woodhouse et al., 2016; de Bont et al., 2019).

This collaborative turn draws literature on social learning into innovation thinking (Colvin et al., 2014; Dessie et al., 2013; Kroma, 2006; Lowe et al., 2019). While drawing on the psychology of learning (Bandura, 1977) and the sociology of learning in groups (Argyris and Schon, 1978), social learning lays emphasis on how culture, context and established practices influence – and are influenced by – the learning that takes place (Lave and Wenger, 1991; Lumosi et al., 2019). As Ensor and Harvey (2015, p.510) summarise, this is a situated understanding that focuses on how “learning emerges from the collaborative processes that allow a shared sense of meaning to be arrived at by the community”. Social learning interventions – structured processes facilitated by external actors – thus offer an approach to managing the interface between differently situated forms of expertise, with learning expanding the boundaries of understanding among those stakeholders who are engaged in a social learning process (Bos et al., 2013). In this view, expertise is itself socially contingent, understood as “the skilful development and deployment of knowledge and other technical capabilities”, that is gained and recognised within social groups or communities of practice, and developed interactively through incremental experimentation and experience (Lowe et al., 2019, p.29). Those designing social learning interventions “focus on enabling new meaning to be found through interaction with those who have a different perspective, in a process of shared ‘sense-making’ around particular issues or challenges” (Ensor and Harvey, 2015, p. 510). Design of interventions is concerned with how interactions can be orchestrated to promote learning, drawing attention to the centrality of facilitation in supporting iterative engagement, openness and trust, and in guiding deliberation that leads towards critical reflection (Colvin et al., 2014; Ensor and Harvey, 2015; Lumosi et al., 2019).

Scholarship connecting social learning and AST innovation includes examples concerned with farming practices (Akpo et al., 2014; Dogliotti et al., 2014; Kroma, 2006; Schneider et al., 2012), decision support systems (Eastwood et al., 2012; Thorburn et al., 2011) and networks of innovation (Beers et al., 2014; Hermans et al., 2013; Oreszczyn et al., 2010; Tisenkopfs et al., 2014; Wals and Rodela, 2014). Changes in practices, understandings and relationships can be identified within this literature ( de Bruin and Ensor, 2018), but the questions of who learns, and what is learnt, lack attention. This oversight is not only found in relation to AST. The failure to analyse learning has formed an important critique of work on social learning interventions generally (Armitage et al., 2008; Baird et al., 2014; Crona and Parker, 2012; Rodela, 2011; Waters-Bayer et al., 2015). In response, an analytical strand has emerged within the social learning literature that aims to unpack the learning that takes place in different settings. The question ‘who learns what, how and when’, is a point of departure of fundamental importance, analysis of which has consequences for intervention design. A focus on learning has the potential to illuminate procedural effects and the distribution of outcomes through analysis of the emergence of new knowledge and shared understandings among differently positioned stakeholders (Bennett and Howlett, 1992; Lebel et al., 2010; Munaretto and Huitema, 2012). The insights from this literature can be used to better understand who learns and what is learnt in farmer-led innovation processes.

Following Huitema et al. (2010), cognitive, normative and relational learning have become an established basis for the systematic assessment of social learning, focusing attention on the forms of learning that emerge (Baird et al., 2014; Fisher and Dodman, 2019; Haug et al., 2011; Huitema et al., 2010; Lebel et al., 2010; Munaretto and Huitema, 2012). As Table 1 summarises, these forms of learning are not hierarchical but, rather, can be independently identified: cognitive learning relates to the acquisition of factual information; normative to changes in norms, values, and beliefs; and relational to the building of relationships, trust and appreciation for different worldviews. Cognitive learning may involve restructuring existing knowledge or the introduction of new knowledge, but inevitably builds on prior experiences and potentially draws on emotions, values or perceptions in the process of interpretation (Röling, 2002; Webler et al., 1995). Normative learning occurs when there are changes to the underlying framework for understanding or interpreting, and is considered by some to be the goal of social learning. Yet as Baird et al. (2014) point out, this potential bias towards ‘deeper’ paradigmatic change may miss the significance of cognitive changes to problem solving and the emergence of virtuous cycles of cognitive learning that themselves lead to deeper shifts (see also: Owens et al., 2003). Relational learning refers to the non-cognitive forms of learning, centred on the appreciation of worldviews and perspectives of others, that in turn can lead to increases in trust and cooperation between stakeholders.

**Table 1:** A framework for analysing learning (adapted from Baird et al., 2014).

Learning type	Characteristics
Cognitive	Acquisition of new knowledge; restructuring of existing knowledge; shifting how situations are comprehended
Normative	Changes in norms; changes in values; changes in paradigms; convergence of group opinion
Relational	Improved understanding of mindsets of others; building of relationships; enhanced trust and cooperation

The starting point for learning lies in dissatisfaction with existing understandings of a situation, which may arise through a “disorientating dilemma” that emerges from exposure to alternative ways of understanding or appreciating a problem setting (Fernández-Giménez et al., 2019; Pennington et al., 2013; Posner et al., 1982). This exposure challenges the frames through which learners interpret and communicate a situation, destabilising how they construct meaning. This challenge may be felt in particular in relation to issue framing, through which actors “define or ‘frame’ a domain as problematic and requiring intervention through selectively identifying the main issues and delimiting its boundaries” (Pahl-Wostl et al., 2007, p. 5). The process of issue framing allows groups of actors to delimit problems by adopting a focus on parts of the issue that may have little or no significance for those outside the group. Collins and Ison (2009, p. 367) refer to the “epistemological constraint” within a learning process that determines what is considered acceptable knowledge or practice in a given setting. However, when the understandings or mental models of learners clash, the resulting “cognitive struggles” can, if addressed constructively, drive a creative process that engages reflection and critical thinking, potentially leading to reframing that augments, overturns or replaces established understandings and mental models (Fernández-Giménez et al., 2019; Mezirow, 1991a; Turner, 2016).

Work characterising learning as multi-loop (Argyris and Schon, 1978; Pahl-Wostl, 2009) is often presented alongside the cognitive, normative and relational framework, helping distinguish between cases where underlying assumptions or frames are challenged (in double or triple loop learning, implying normative and relational shifts) or left in place (single loop or cognitive learning). Reed et al. (2010, p. 3) distinguish between “learning about the consequences of specific actions” and “reflecting on the assumptions which underlie our actions” in single and double loop learning respectively, while triple-loop learning “challenges the values, norms, and higher order thinking processes that underpin assumptions and actions”. As they point out, social learning may occur at any of these levels (Reed et al., 2010) and can be identified in terms of individual, network or system level change (Rodela, 2011). While cognitive, normative and relational learning can occur independently within learning processes (Baird et al., 2014), interconnections may also emerge. For example, the process of critical reflection and challenge embedded in double and triple loop learning suggests a connection between the normative and relational aspects of social learning, where stakeholders are brought together so they “can learn from and about each other”, unpacking assumptions as their understanding and respect for alternative viewpoints builds (Lebel et al., 2010 p.348; Pahl-Wostl, 2009). While there is growing evidence that relational learning may allow for the emergence of cognitive and normative learning (Fernández-Giménez et al., 2019), Baird et al. (2014) suggest that normative learning is harder to access than cognitive or relational shifts, not least because attitudes and norms are slow to change across a range of contexts (Roland, 2004; Williamson, 2000). McFadgen and Huitema (2017) draw attention to the influence of how the process of engagement is designed in influencing what is learned. They suggest a trade-off between cognitive, normative and relational learning embedded in the emphasis placed on building particular aspects of “objective” knowledge among stakeholders, versus the emphasis on a participatory approach that draws out and values alternative expertise. Thus, time and effort spent supporting the emergence of shared values and new norms may be at the expense of stakeholders acquiring new knowledge (and vice versa). In the following, the analytical framework of cognitive, normative and relational learning is used in the analysis of a famer-led innovation action research project.

## 3 The action research process and methodological approach

This paper reports on a farmer-led innovation process that was convened to explore the potential of, and draw lessons from, placing farmers at the centre of a structured process of defining problems connected to farming practice and identifying and co-developing potential AST solutions.

### 3.1 Two case studies

An open recruitment campaign was run via media, partner organisations and local farmer networks, inviting farmers to join a group in which they would “identify the challenges that need to be met on their farms, and to get involved in designing and testing new technologies that may be able to help” (unpublished project publicity material, 2017). While the understanding of the term ‘innovation’ that grounded the group process included changes to processes, institutions and ways of working (see Section 3.2 below), advice from project partners recommended that recruitment should be based around simple messages and focus on the potential for technology development. The farmers were brought together in one of two groups, analysed in the following as two case studies: one in northern England (the NE group) and one in the Scottish Borders (the SB group). The NE group consisted of eight mixed farmers who were separately recruited to the project. The SB group was made up of the staff responsible for running farming activities on part of a large estate. Half of the SB group specialised in sheep husbandry and production (referred to here as shepherds) and half were focused on cattle (cattlemen). The farm manager, who linked the shepherds and cattlemen to the wider running of the estate, was also present at most meetings. In the following we refer to the farmers, shepherds and cattlemen as ‘farmers’, unless there is a particular need to distinguish between these sub-groups.

Following recruitment, the action research process was premised around the potential for improvements in farm livelihoods and wellbeing through novel applications of AST. This starting point recognised that technical changes are anchored in and influence the social and institutional context. To support this process, in both case studies the same engineer and facilitator were present at all meetings alongside the farmers. The engineer was recruited for the project and based at a partner university. This provided him with direct access to a team of applied scientists working on different aspects of AST within a large engineering department, as well as to a wider network of scientists. The engineer was positioned within the group as being at the disposal of the farmers, functioning as a conduit between the farmers and other scientists. The facilitator – this paper's second author – had primary responsibility for convening and running the group meetings, managing relationships and, together with the lead author, designing the meetings and analysing the process and emerging outcomes.

### 3.2 Learning process design

The structured process consisted of a series of meetings carried out during 2017–2020. Together these meetings were designed as a learning process, in which the farmers, engineer and – when identified as needed by the participants – wider stakeholders were able to learn together about problems and potential solution pathways. The overall learning process design was conceptualised as a social learning intervention. In common with Lumosi et al. (2019, p. 68), each meeting was intended as “an arena for interaction, deliberation and re-framing”, leading to a series of facilitated spaces in which the groups were brought together over a three-year period with the intention of enabling new understandings to emerge. Meeting design thus focused on interaction (communication and exchange of information), deliberation (allowing different viewpoints to be exchanged and reflected upon) and re-framing (opportunities to question assumptions and enrich understanding by building on others' frames) (Lumosi et al., 2019). In the design of the group meetings, innovation was defined as “new processes, institutions or ways of working that aim to meet a set of needs or tackle a set of problems” (Colvin et al., 2014, p. 761), and in keeping with the ethos of democratisation of AST, was understood to emerge through a change in understandings or practices that is both grounded in and gives rise to changes in relationships (cf. Collins and Ison, 2009; Klerkx et al., 2010). These changes create space for innovations to emerge that are responsive to context and respectful of a plurality of knowledges embedded in differing epistemological backgrounds.

Process design centred on the development of facilitation guides for each meeting of the group, with the emphasis being on securing opportunities for the emergence of new understandings. The task for the facilitator was thus to support group co-ordination and momentum, and the opening of spaces for interaction, deliberation and critical reflection / reframing. This leads to what Colvin et al. (2014, p. 767) refer to as a “part preconceived, part emergent” design praxis: creating space for dialogue while working within project time constraints and guided by an overarching goal of enabling new understandings to emerge in relation to on-farm problems. Significantly, the overall process design proceeded from the understanding that to open discussion spaces without explicit recognition of power and context would be to invite the reproduction of past experiences of innovation. Multiple studies underscore the potential for power relations between stakeholders, and for established norms and practices, to be reinforced through participatory processes in general, and co-production and social learning approaches in particular (Ensor and Harvey, 2015; Goldman et al., 2018; Muro and Jeffrey, 2008). The farmers in the groups in particular had long association with the dominant model of AST, having been adopters of technologies over many years. Similarly, the scientists whom the engineer provided access to worked within a setting with well-established technology development norms and practices.

**Fig. 1:**
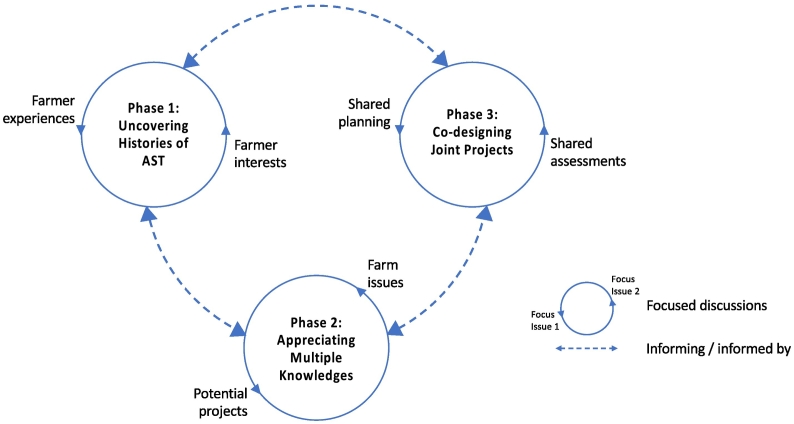
The three interconnected phases of the learning process and the focus of discussion in each phase.

The first phase of the learning process thus invited the farmers to reflect on their experiences of AST, with the groups documenting their perceptions (positive and negative) of the trajectory of technology change and how this had interacted with their motivations and interests as farmers. This exercise, referred to as the first phase of the learning process (‘Phase 1: Uncovering histories of AST’ in Fig. 1 and Table 2), aimed to support critical thinking and was referred back to over the course of the learning process to encourage the farmers' confidence in the legitimacy of their knowledge and experience of farming and AST. These sessions thus had a normative goal, deliberately looking to foreground tacit knowledge and counteract farmer experiences of exclusion from mutually reinforcing knowledge, power and sense-making practices that are inherent to the dominance of contemporary AST. During these opening sessions the engineer's role was as an observer; his active participation in the dialogues gradually increased thereafter.

**Table 2:** The phases, purpose, methods and participation in the farmer-led learning process meetings.

Phase of the learning process	Purpose^	Methods^	Who*	Meetings	Period
Pre-meetings	To meet and get to know farmers and their context.	Walking interviews across each farm.	F, Fac Sh, Fac Ca, Fac	Farmer meetings	Nov 2017
Phase 1 Uncovering histories of AST - Farmer experiences and interests	To establish the group. For the famers to reflect on their experiences of AST. To identify farmers' interests.	Association exercise on farmer wellbeing. Clustering exercise of individual positive and negative experiences of histories of AST. Listing potential issues to work on and initial voting to prioritise.	F, E, Fac M, Sh, Ca, E, Fac	NE1 SB1	Nov 2017
	To agree on shared set of statements about what ‘innovation’ should do. To prioritise a subset of the issues identified for further development	Summary of first meeting. Group discussion about what innovation should do based on histories of AST and farmer wellbeing Multi-criteria analysis of all the issues identified in the first meeting with criteria identified by the farmers.	F, E, Fac M, Sh, Ca, E, Fac	NE2 SB2	Feb 2018
Phase 2 Appreciating multiple knowledges - Potential innovation projects and farm issues	To gain a deeper (spatial and temporal) understanding of the prioritised issues. To list criteria against which potential solutions should be assessed.	Farming maps; farming calendars. Group discussion about why technologies or practices currently used are not sufficient. Group discussion about what innovation should do, based on Phase 1 themes.	F, E, Fac M, Sh, Ca, E, Fac	NE3 SB3	Mar 2018 June 2018
	Engineer identifies potential innovation projects.	Meetings with scientists working on projects allied to group interests and reviewing literature.	Exp-S, E		
	To reconnect with the process after the break. To inform farmers about the potential innovation projects.	Revisiting group discussion about the Phase 1 themes. Engineer introduces potential projects; group relates projects to their farm issues. Questions identified to inform engineer priorities for next meeting.	F, E, Fac M, Sh, Ca, E, Fac	NE4 SB4	Nov 2018 Dec 2018
	To agree on list of themes related to what innovation should do. To update the farmers on the potential innovation projects and get feedback. To prioritise innovation projects.	Revisiting and agreement on Phase 1 themes. Engineer update on each of the potential projects. Agreed Phase 1 themes incorporated into a multi-criteria analysis to shortlist potential projects.	F, E, Fac M, Sh, Ca, E, Fac	NE5 SB5	Dec 2018 Jan 2018
	To update the farmers on the shortlisted innovation projects and get feedback. To agree on which innovation projects to take forwards. To allocate the engineer's time to each innovation project.	Engineer provides detailed update on the shortlisted projects which the group discusses. Individual perspectives on project(s) to take forward are discussed; final decision made. Farmers decide on the allocation of the engineer's time on each project.	F, E, Fac Sh, Ca, E, Fac Sh, Ca, E, Fac	NE6 SB6 SB7	Jan 2019 Feb 2019 June 2019
Phase 3 Co-designing joint projects – Shared planning and shared assessments	To update the farmers on the shortlisted innovation projects and get feedback. To bring experts in to further the understanding of the innovation project or the institutional context.	NE-group: A scientist joins the meeting to present one of the innovations in more detail. The group discusses with him. SB-group: A meeting brings the group together with the health and safety officer of the estate and a manager of another part of the estate. Farmers present their perspective; group explores the institutional context of lone worker safety across the estate.	Exp-S, F, E, Fac M, Sh, Ca, E, Fac Exp-H, Exp-M	NE7 (SB) LWS	Mar 2019 Mar 2019
	Engineer further develops the shortlisted innovation projects.	Meetings with experts and technical development. NE-group related: A visit to a veterinarian to further understand practices related to undertaking on-farm diagnostics. SB-group related: Farm visit to the SB group to understand the physical context. In these meetings the facilitator undertakes participant observation.	Exp-V, E, Fac Ca, E, Fac	(NE) Vet visit (SB) Farm visit	July 2019 Mar 2019
	To co-design the innovation projects iteratively. To test the innovation projects in context on farm. To reflect on progress made in relation to the themes related to what innovation should do	Farmers are provided with the innovation projects and given an update from the engineer. Co-design of the innovation projects through group discussion and experimentation. Iterations between the technical development by the engineer and the assessment of applicability in context of the farmers. Group discussion about the progress made in relation to the Phase 1 themes.	F, E, Fac M, Sh, Ca, E, Fac M, Sh, Ca, E, Fac	NE8 SB8 SB9	Nov 2019 Oct 2019 Nov 2019
	Joint farmer group meeting to hear about the innovation projects. To reflect on progress made in relation to Phase 1 themes. To reflect on the learning process.	Each group presents their innovation project, with support from the engineer. Each is discussed. Group discussion about the progress made in relation to Phase 1 themes. Initial individual reflections and group discussion structured around themes related to cognitive, normative, and relational learning.	F, Sh, Ca, E, Fac	Shared group meeting	Dec 2019
	To provide an update on the innovation projects of both groups. To identify next steps with the experts.	NE group: The engineer and expert updates the group on the final iteration of the innovation projects. The group gives a final round of feedback reflections. SB group: The health and safety officer is updated by the farmers. The group gives a final round of feedback reflections. Steps to change the institutional context are identified.	Exp-S, F, E, Fac Exp-H, Sh, Ca, E, Fac	NE9 SB10	Feb 2020 Feb 2020
Closing meeting	To provide the final update on the innovation projects of both groups. To reflect on the learning process. To identify next steps if relevant.	The engineer provides a final update. Further individual reflections and group discussion structured around themes related to cognitive, normative and relational learning.	F, E, Fac Ca, E, Fac	NE10 SB11	July 2020 May 2020

^the word ‘farmers’ refers to the farmers in the NE group and the shepherds and cattlemen in the SB group.

^⁎^Farmers (F), Shepherds (Sh), Cattlemen (Ca), Farm manager (M), Engineer (E), Facilitator (Fac).

Experts: Scientists (Exp-S), Health and safety officer (Exp-H), Manager of another part of the estate (Exp-M), Veterinarian (Exp-V).

After these opening meetings, multiple methods (see Table 2) were used in the second phase to build understanding of the different farming contexts and to explore the issues the farmers wanted to work on (‘Phase 2: Appreciating multiple knowledges’). The engineer worked between the meetings to review the issues and network with specialists in his university, enabling him to present a number of potential ‘innovation projects’ the groups could work on. Through an iterative process of information gathering, reflection and prioritisation, the farmers and engineer discussed and ultimately selected the projects they wanted to take forward (‘Phase 3: Co-designing joint projects’). These projects were then developed in the third phase through an iterative process that involved successive rethinking of the issues in light of the developing project, and the project in light of the issues. At each step, facilitated discussions supported the farmers and engineer to develop priorities for further design iterations. In total the NE group met 10 times and the SB group 11 times. At the midpoint of the final year and at a closing meeting the groups were brought together to hear about the projects each group had worked on, during which sessions were also structured to elicit farmer and engineer reflections on what had been learnt in terms of cognitive, normative and relational learning. Table 3 provides an overview of the issues identified and the innovation projects prioritised in each group.

**Table 3:** Innovation projects identified by the two groups in response to issues identified during the first phase discussions.

Initial issues	Shortlisted innovation projects	Final status of projects
NE: Being proud of livestock and livestock products SB: Recording of medical and veterinary data	• Livestock database phone app: Intended to allow simple collection and retention of important data on individual animals. The NE group decided to develop this. The SB group chose to focus only on the lone worker safety app.	• App development completed; app installed and used by some of the farmers. No further dissemination activities during the project.
SB: Lone worker safety	• Lone worker safety phone app: This innovation had two components. The group decided to focus on making this work in the shed for the cattlemen, but with the intention that it could be adapted in future to work for shepherds on quad bikes in the remote hills.	• App development completed and used in practice by the cattlemen. Institutional changes made to ensure follow up in case of an emergency. App profiled in national media and uploaded to an app store for further dissemination.
NE: Targeted interventions	• Sampling and detection: This issue evolved into two projects: a leaf mimic, which allows farmers to test for the presence of crop pests or diseases; and an on-farm blood sampling device for use with livestock.	• Leaf mimic undergoing further development at the end of the project.. • Mechanics for blood sampling tool developed by the group now used as the basis for ongoing research and development of rapid tests for diseases in animals.

	Innovation projects not shortlisted	
NE: Soil management	• Mobile methods to test for greenhouse emissions in the field • Measuring soil carbon • Measuring soil diversity • Developing a knowledge-based to better manage soils	• Not progressed
NE: Being proud of our produce	• Exploring ways to connect farmers with food banks to reduce waste	• Not progressed
SB: Animal monitoring	• Mechanisms to automatically send animal data to a centralized system	• Not progressed
SB: Recording livestock	• Novel ways to find animals obscured from view in remote landscapes	• Not progressed

The overall learning process design was the same for each group, with each meeting in the structured process working to a common agenda - although adapted to the issues and innovation projects that emerged. Both groups had experts join the conversation (see Table 2). In the SB group a health and safety officer and a manager of another part of the estate joined, and in the NE group a sensor scientist specialising in bio-electronics joined the conversation. A veterinarian also provided feedback on the on-farm blood sampling device in a separate meeting.

### 3.3 Data collection and analysis

With the permission of participants, the meetings and interviews were recorded and transcribed verbatim, supported by field notes made by the facilitator that in particular captured body language and significant moments of interaction. Alongside the transcripts of the workshop meetings, data was gathered during an initial farm walk, in follow-up interviews after the workshops, and in a closing meeting. The latter provided space for farmer reflection on their experience of the learning process overall. The transcripts and notes were subject to a broadly deductive thematic analysis, aided by NVivo analysis software and informed by our reading of the social learning literature (Braun and Clarke, 2006). Coding was used to organise data, with initial codes identified from the literature, as set out in Table 1. That is to say, our interest in coding was not in providing a rich description of the entire dataset, but rather to focus on aspects connected to our overall interest in describing forms of learning that took place. The following section sets out the themes that recurred in relation to cognitive, normative and relational forms of learning, describing how these emerged within the structured learning process. The discussion then looks across these examples to understand the interconnected mechanisms that allow these three types of learning to emerge within an overall process, drawing lessons for future practice.

This approach to data collection placed a dual researcher-facilitator role on the second author, complicating her remit within the learning process. While the recruitment of a dedicated facilitator would have allowed the researcher to operate in a purely observational mode, the dual role allowed for the distance between group members and the researcher to be minimised, allowing valuable information to be collected during the project activities (Akpo et al., 2014). As Ensor and Harvey (2015) note, researchers are often well placed to convene stakeholders, provide feedback to aid learning, and to support the emergence of documented social learning processes and outcomes. This dual role does not, however, come without challenges. The researcher-facilitator is required to engage deeply with the problem setting, with the goal of facilitating learning among those who may initially be resistant and whose experiences will need to be addressed with empathy, humility and care; meanwhile the practical challenges of providing effective and flexible facilitation that is sensitive to group power dynamics while securing data suitable for research outputs are very real (Bardsley, 2014; Herbert, 2010). The potential for bias or selectivity in the voices foregrounded in analysis was reduced by working with transcripts and a rigorous approach to coding, undertaken in collaboration with the first author, after the completion of the project. This helped to secure distance for the research role, while the use of multiple methods (meeting transcripts, interview notes, diagrams, participant observations) provided opportunities for triangulation, and the joint analysis with the first author provided a degree of external perspective.

Inevitably, working in this way also challenges the incentives and goals of research professionals that are, for the most part, orientated exclusively towards securing publishable outputs. In our project, we aimed from the outset to place decision making over the innovation process into the hands of the groups, leading to “a necessary eventual marginalisation of the researcher-facilitator from many of the core outcomes of the research process” (Bardsley, 2014, p.43), reflected in practice in the increasing autonomy of the groups as the project progressed.

## 4 Results

In the following, direct quotes are attributed to a farmer (F) Shepherd (Sh), Cattleman (Ca), Farm manager (M), or engineer (E) in either the northern England (NE) or Scottish Borders (SB) group meetings, where each group meeting is numbered (see also Table 2). Where expressions or phrases were frequently repeated in the meetings, they are not provided with an attribution in the text. We refer throughout to members of both the NE and SB groups as ‘farmers’, reflecting their similar role as skilled, knowledgeable practitioners and empowered decision makers in both settings. Further disaggregation is offered where required to explain aspects of the results.

### 4.1 Cognitive learning

Evidence of cognitive learning emerged from two main processes, both of which provided moments where the groups were exposed to new information or developed a new appreciation of their situation. The first was during instances of framing and reframing the issues under consideration, illustrated here in relation to soil health and lone worker safety discussions. The second – making sense of innovations – occurred in three broad steps: clarification of new information, contextualisation of that information, and co-design of the innovation projects.

#### 4.1.1 Framing and reframing

Although not progressed as a project, the NE group initially identified soil health as an important issue. First framed as a sustainability issue (“leaving the land in a better way than we found it”; F1, NE3), the farmers discussed how they were fundamentally dependent on their land, and were acutely aware of the need to improve soil health if next generation of farmers were to be successful. Discussions focused on farm practices that caused soil damage, gradually leading to a reframing of soil health as a knowledge issue: farmers were interested in furthering their own understanding of soil health and to validate their experiential knowledge (“Well, we know we don't really know that much about soil.” F5, NE4). Several farmers expressed a desire to understand the soil better in order to “get the best out of it” (F5, NE4). Iterations of these discussions included input from the engineer on compaction, greenhouse gas emissions, soil carbon and soil diversity, progressively expanding the boundaries of the issue under discussion. Understanding was built as the farmers reassessed their appreciation of soils in their diverse settings, and expanded their knowledge of soil science and of the potential and limitations of alternative soil testing methods and approaches.

In the SB group lone worker safety was identified as an important issue at the first meeting. At the time it was framed as a technical issue in relation to the frustrations staff experienced with their existing system: “it doesn't put out a signal at all when you're in a shed.” (Ca1, SB1). This technical framing remained present during the co-design process as the group aimed to address the issues related to signal coverage inside a shed and in some upland locations. Farmers also reflected on the issue as a personal experience. They discussed when they had felt at danger, and shared stories of others who had come to harm whilst working alone. The group came to understand lone working as an issue affecting the agricultural sector as a whole and significant for both shepherds and cattlemen in the group. However, the shepherds agreed that the cattlemen were more at risk than them: “these boys are often at night [by] themselves.” (Sh1, SB5). Appreciating lone working as an issue of personal significance led to it being prioritised over other issues identified by the group. The importance of this framing also led the group to actively engage with potential solutions outside of the structured process of the meetings. A third frame was put forward by the farm manager and later by the estate health and safety officer when he was invited to join the group: they presented lone working as an institutional issue and the responsibility of the estate to look after its staff with health and safety processes. By bringing the health and safety officer into two meetings, the group was able to consider and thus ultimately navigate between three frames that enabled a wider, more systemic understanding of the problem to emerge. Solutions were thus sought that would make staff feel safe; that would work technically in a complex environment; and that provided institutional protocols if a person was identified as at risk or in the process of coming to harm.

#### 4.1.2 Making sense of innovations

In the second and third phase the engineer provided an update about what information he had accessed about the issue or innovation projects since the last meeting. In this he referred to previous discussions and questions set by the farmers, and relied on findings gathered from scientists at his host university and/or a review of published and grey or commercial literature. Examples of other technologies or practices that the farmers might recognise were used to set out scenarios and consider potential outcomes. Through discussion, the farmers made sense of the innovation projects and deepened their understanding of the technical and scientific considerations often relating to other tools or technologies they were aware of (with phrases such as: ‘Is it like…?’) and imagined what the possibilities could be (‘Can it …?’). At times the farmers made assumptions that were not possible or offered suggestions which would take too long to develop; in these cases, the engineer intervened in the conversations (‘It can't do that’; ‘That wouldn't work, because…’). Any questions that emerged as significant to understanding were investigated by the engineer in the period between the group meetings. Over the course of the meetings farmers learned enough about the innovation projects to at times speak for the engineer and explain to other farmers in the group what it entailed, what its limitations were, and how long it took to develop.

Farmers subsequently contextualized the innovation projects and thought through the practicalities in relation to their own practices and farm setup. Farmers were concerned that innovations be practical (for example, “Is it ‘Sumo-proof’?”, meaning physically strong enough to cope with the farm environment^1^), work in any type of weather (‘fifty mile an hour winds’ or ‘chucking it down with rain’), easy to use (‘not create more work’), and fit within their current practices (‘I always have a phone in my pocket’). Whilst thinking through what the innovations could do, farmers also reflected on what should be avoided, based on their experiences with current and previous technologies. This often related to negative experiences (for example, databases need to ‘talk to each other’). These experiences were often shared by other farmers in the group, in turn reinforcing understanding and refining the design attributes. This process of contextualising thus led to moments of co-design between farmers, the engineer, and other experts who were present. As part of this process not only were the innovations further developed, but the farmers also continued to develop a shared image of what these innovations could help them do, further refining the focus and ambition of the projects. Often this included potential learning outcomes they could achieve: ‘With that information I could…’. In the SB group, for example, the meetings resulted in a design feature to adjust the lone worker phone app so that it can detect a fall irrespective of where the user keeps their phone: “Just cos we've had these meetings we've kinda made that difference; so whereas the shepherds normally have it [their phone] in their breast pocket, that we have it in our, well jeans pockets, …on the app you can put that in…” (Sh2, SB10).

### 4.2 Relational learning

Evidence of relational learning emerged in all of the meetings, in the different ways that participants related to each other and worked together, and in how the position of actors in the groups changed over the course of the process.

#### 4.2.1 Relating to each other and working together

In both groups the farmers had not met the facilitator or the engineer before joining the process. The farmers in the NE group knew about each other due to their involvement with local and regional farmer groups (e.g. National Farmers Union) and some were already friends. Farmers in the SB group knew each other from working together on the estate. While not all were close at the outset, in both groups there was evidence of a strengthening of relationships when participants referred back to challenges mentioned by the other group members, or demonstrated an interest in the concerns expressed by other participants. Participants displayed openness to each other throughout the process. They were willing to discuss their practices and shared mistakes they had made, encouraged by others who responded with recognition or with an understanding of the challenges faced. The NE group had a supportive dynamic with farmers at times bringing into the conversation others who hadn't contributed. When there was disagreement between farmers they negotiated between themselves and only occasionally needed facilitation to ensure different viewpoints were recognised or that an agreement was reached. In the SB group the dynamics changed depending on whether the farm manager was present at the meetings. This was evident in how much the other participants talked: when the manager was present, he would often respond to questions. However, the other participants were able to increasingly take charge of the process, for example by explaining to the farm manager what had been discussed in previous meetings.

Through working closely with an engineer, the farmers gained an appreciation for the innovation process. The engineer often tried to ensure no one was under any misconceptions about the progress the group could make during the time that was available. “So I can't guarantee the list of things it can do” (E, NE5). In turn, the farmers recognised that developing innovations took time, that “things don't happen just like that, it's a lifetime really” (F3, NE5). The engineer shared some of the obstacles he had had to overcome and was open about things that turned out not to be possible. Farmers gave feedback and validated his approach. “F3) Very practical these experiments so far. E) Well, thank you, like I have tried to make them something you can get… F3) well to be fair you have done that all along, you have mapped it out.” (NE5). Farmers in the SB group all agreed they had gained an appreciation for the complexity of the design process and for the work of engineers; the engineer, on the other hand, reflected how working together with the farmers had opened his eyes, expressing his surprise at many existing challenges, such as when learning that new technologies for lone worker safety hadn't addressed the challenges of working in a shed.

#### 4.2.2 Positionings in the groups

The design of the meetings enabled the researcher-facilitator to facilitate group dynamics between the farmers, engineers and other invited experts. The aim was to give the farmers control, which it was clear from discussions contrasted with their prior experiences of technology development. The engineer was positioned at their disposal. During the first phase the engineer was asked to take on the role of listener. The farmers were encouraged to share their experiences of technologies on farm, and challenges they faced which could potentially be overcome through an innovation process. In this phase the farmers were asked to prioritise the issues identified to ensure the process remained farmer-led. In the second phase the engineer's role became more prominent, feeding back on potential innovation projects. This positioned him as an expert, although at times he explicitly presented himself as a conduit between the farmers and others with particular areas of expertise. Throughout this phase the engineer reiterated that he was at the disposal of the farmers. In the third phase, at the end of each meeting the farmers planned the work of the engineer by deciding what proportion of the engineer's time they wanted the engineer to spend on each of the shortlisted projects. This allowed the farmers to further enact their leading role.

The design of the meetings offered a structure within which farmers, engineers, and experts performed certain roles, but shifting power dynamics also emerged through the process. During meetings with the NE group different farmers took the lead at different stages depending on their interest in the innovation being discussed, or because they were trying to help the facilitation of the process. There was no single individual who dominated. In the SB group the farm manager dominated the conversation when he was present. The group had agreed to prioritise the lone worker safety of the cattlemen, which meant that the shepherds in the group had less to contribute during the second phase. In the LWS meeting the group was expanded to include the health and safety officer and a manager from another part of the estate, with the intention of using the facilitation process to disrupt the hierarchy that had emerged. However, these dynamics were resistant to change, for example with the senior staff leading the discussion and referring generically to the staff sitting in the same room as them. On the other hand, farmers in both groups were confident from the beginning in correcting the engineer when he had made wrong assumptions about certain farming practices. The farmers noticed how well the engineer listened to their ideas and took their questions forward to the next meeting. The farmers continued to drive the process, clarifying the reasons why some projects ended up being shortlisted and others not (“Out of all the projects you've told us about, it [one particular project] might be the most exciting, but it's just practically not there.” F3, NE5). On other occasions the engineer would assert his viewpoint by saying that it was better to focus on one thing and do that well, than to try to do several things. Here, the farmers agreed, recognising this as a potential challenge from within their own experiences (‘we know what it's like’), and ultimately not wishing to see the engineer diverted onto too many tasks. These passages highlighted not only the distinct roles of the participants, the confidence of each to express their views, but also the emergence of a sense of shared challenges between the farmers and engineer.

### 4.3 Normative learning

Normative learning was evident when participants discussed the underlying rationales and values related to agricultural innovation. This focused on two themes: what innovation can and should do, and how innovation can and should be done. The first is illustrated by looking at how farmers identified key attributes of potential technologies, and the second in facilitated discussions reflecting on histories and experiences of AST.

#### 4.3.1 What innovation can and should do

The group discussions developed a shared sense of farmers' experiences of past AST and what this had pushed them towards. They identified efficiency and productivity as a driver that had pushed towards increasing size of machinery, which in turn affected the size of their farm, creating what they identified as a vicious cycle. “We've been channelled and focussed to making our equipment, our time do more. We are psychologically programmed that if you've got a big machine it needs to do more acres. [..] so ultimately we are programmed to run to stand still; that's how we work isn't it?” (F1, NE4). “We've had similar thoughts, yeah. […] we have rented land and doing it for nowt sort of thing and, but yeah, like you say, it's, you want to spread the costs of your machinery but then you go and buy new machinery …” (F4, NE4). On the other hand, the farmers also recognised that this increase in size of machinery was partly in response to increasingly limited windows of weather related opportunities. “In a month there can be probably four or five days where actually you can get on and spray, cos it can't be after rain, it can't be windy, it can't be stonking hot, you know. There's lots of variables we can't do anything about, so hence the investment and the size of the equipment to do the job.” (F6, NE6).

The farmers saw their involvement in the learning process as an opportunity to think about what new technologies should allow them to do, and to contribute to a wider debate about the direction that farming is going. Most importantly, they felt that new technologies and processes should allow them to make better decisions, by gaining additional knowledge about their farm, their soil, animals and crops. This was framed in terms of being able to ‘see the invisible’: “F2) You might not see it with the naked eye, but the machine can and so you can do something before it gets to the stage where it is too late.” (F1, NE6). Information about the invisible could also enable farmers to validate their experiential knowledge. “We're all fairly experienced, you do know if there's gonna be a bit of a septoria outbreak, you know the rainfall, you know what triggers it off, and the same as the flea beetle and all things like that, and then you maybe go… [to the technology] to confirm.” (F3, NE6). In these conversations the vision was for technology that supported the farmers' decision-making processes. It remained important for farmers to go out to the field themselves “[machinery] ain't gonna be as reliable as you going out and do it yourself.” (F6, NE6). In the SB group they took this even further saying that it was critical that you had better stockmanship than a robot: “if your technology's telling you what's wrong you still need a stockmanship to be able to stand and interpret that data and, and actually look at the animal and say, yeah, that's right, that is wrong.” (M, SB4). The discussions in both groups moved towards an understanding of the role and purpose of innovation as contributing into a decision support system that would allow them to take better care of their soil, animals and crops.

#### 4.3.2 How innovation can and should take place

In the first phase of the learning process, the facilitator invited the farmers to discuss ‘why do we innovate?’, and regularly reflected on this discussion as a way of keeping the overall process focused around the farmers' underlying priorities. Analysis of these wide ranging discussions revealed a number of themes related to what innovation should do: contribute to profitability; fit within different contexts and therefore be relevant to everyone in the group; be practical; contribute to farmers' feelings of pride and a sense of progression; and to help farmers make better decisions. When the farmers were presented with this analysis they reflected: “F1) You've turned this all on its head because ultimately when we go and make purchasing decisions we walk around something which is new, we rub our chins, we pull our hair out and decide how we're off to pay for it, and we just jump, and actually it's really weird listening to you taking this completely the other way around. We are very accepting of other people's innovations. It's whether we're prepared to buy into it. That's how we've always performed. F5) that's right, it's been sort of pitched to us, hasn't it…F2) and whether you'll take the risk or not… F1) And this is really surreal (laughter).” (NE5).

The farmers were at times somewhat confused and surprised by being listened to. They reflected on how experience had eroded their sense of place and ownership in the agricultural innovation processes, contrasting the group work with other experiences: “We could say we want to have this, this and this, instead of getting something from DEFRA …” (F1, NE5). When asked what they thought about the livestock database app once they had access to it during a meeting: “I think it's really nice and sort of strange that somebody's made something for us as opposed to accepting whatever shit we usually have to purchase or get. It's a totally different way around of thinking” (F1, NE7). They recognised that the engineer had taken into account what the farmers had discussed in previous meetings. “It's what we've asked for, which is quite a thing. And I do think the simpleness of it is a massive thing really. [..] That was one of the main things that kept coming up [in our discussions].” (F3, NE7). Both groups appreciated this rethinking of the innovation process when reflecting on their experiences of the group. As one farmer reflected: “It's no use bringing it [i.e. high technology] all the way here and it falls down at the practical end [on the farm].” (F3, NE6). In the SB group this was recognised as well. “Just by having these kinda meetings …. It's helped us come up with this … everyone's got their own ideas and everyone works in certain ways so they put forward their opinions and you can kinda alter the app that way.” (Sh2, SB10).

## 5 Discussion

Designed around social learning principles, the group meetings in this farmer-led innovation process created space for sense-making around issues and potential technological solutions, providing an arena in which new meaning could emerge (Lumosi et al., 2019; Steyaert et al., 2007). Participants acquired new knowledge and, in a process of framing and reframing the issues and potential technologies, negotiated meaning that led to a shift in how they understood their situations (Pahl-Wostl et al., 2007). Not only were participants able to make sense of themselves and their own context, they also related to each other and negotiated the meaning of the farmer group situation, the issues and innovations, and what innovations can and should allow farmers to do. These shared, contextual realities emerged as they negotiated different, although at times overlapping, frames of farming practices and technologies (Dewulf and Bouwen, 2012; Pahl-Wostl et al., 2007). As the meetings built on from one another and included references to previous meetings, these frames and shared realities continued to evolve over the course of the process. Cognitive, normative and relational learning emerged within the facilitated structure of the meetings, and were frequently interrelated. As summarised in Table 4, the three phases provided opportunities for learning, albeit within a process that progressed iteratively and regularly referred back to, revisited or revised the conclusions of earlier phases.

**Table 4:** Learning in three phases of the farmer group discussions.

phase of project	EVIDENCE OF LEARNING			OUTCOME of Phase
	COGNITIVE	NORMATIVE	RELATIONAL	
Phase 1: Uncovering Histories of AST *WHO: FARMERS* *WHAT: REFLECTING ON EXPERIENCES AND INTERESTS*		• Self-reflection on motivations for farming • Critical analysis of experiences of technologies • Developing new shared norms	• Recognising shared experiences • Developing new shared norms	• Shared view of purpose and value of AST • Initialised group relationships and practices of critical engagement • Identification and prioritisation of farmer interests
Phase 2: Appreciating Multiple Knowledges *WHO*: *FARMERS, ENGINEER* *WHAT: IDENTIFYING ISSUES AND POTENTIAL PROJECTS*	• Sense making • *Re*-framing • Merging frames	• Rethinking the purpose of technologies • Imagining what could be done	• Appreciation of each other's understandings and practices	• Shared understanding between farmers of their contexts and challenges • Knowledge exchange between farmers and engineers • Identification and prioritisation of innovation projects
Phase 3: Co-designing Joint Projects *WHO*: *FARMERS, ENGINEER, EXPERTS* *WHAT: SHARED PLANNING AND ASSESSMENT OF PROJECTS*	• Deepening understanding of technologies	• Rethinking the purpose of technologies	• Undertaking shared assessments of projects	• Design, development and testing of technologies • Deeper, shared view of the purpose and value of AST • Confidence of farmers and engineer to work together independently of facilitation

Normative questions were explicitly raised in the first phase of group discussions, in which facilitation centred on histories of AST and asked farmers to reflect on what they hope to gain from innovation. These discussions informed the subsequent group meetings and invited the farmers to engage with their experiences of the advantages and drawbacks of AST, in the context of their different motivations and aspirations as farmers. The farmers shared their own experiences and often agreed with each other, recognising familiar patterns of where new technology had improved or further complicated their lives and livelihoods. This process allowed the farmers to move together towards a new understanding of the problem of AST, but at the same time was disorientating. It was important in later meetings to return to the analysis undertaken by the farmers during this phase to stop conversations from falling back into more familiar patterns of presumed advantage associated with scaling-up and time efficiency. In a similar vein, at different times engaging with their own assessment of AST priorities was met with laughter or described by the farmers as a “surreal” experience of thinking differently (NE5).

These forms of unease or discomfort can be a catalyst for learning: the disorientating dilemmas that can pave the way for later phases of cognitive, normative and relational learning (Baird et al., 2014; Fernández-Giménez et al., 2019; Keen et al., 2005; Mezirow, 1991b). In the first phase, this disorientation arose not from a clash of preconceptions among the stakeholder group, but from grounding the learning process in the starting conditions that had given rise to the current situation faced by farmers (Colvin et al., 2014; Collins and Ison, 2009). Thus, focus in the groups on the significance of context not only orientated discussions towards the genesis of current challenges, but also set the stage for learning by exposing dissatisfaction with existing understandings and practices (Posner et al., 1982). In this way, this phase of the farmer groups differed from many examples of social learning, in which learning is predicated around resolving divergent perspectives on a common problem (e.g., Cuppen, 2012; Ensor and Harvey, 2015; Morris et al., 2020). As the farmer groups moved into the subsequent phases, learning was supported by a focus on dissatisfaction associated with practical problems, and different understandings arising from different forms of expertise. However, our results suggest that it was the backdrop provided by the discussion of AST history and context, and the subsequent opportunities for reflection on the success and failure of current innovation practices to meet farmers' needs, that precipitated a normative reimagining of AST innovation that was ultimately shared by the farmers and engineer in the group (Pennington et al., 2013).

This process of self-reflection and critical analysis was intertwined with relational learning, experienced as the emergence of a new, shared vision for the process and purpose of AST innovation and a deepening of understanding and trust among the group members. This interconnection between normative and relational learning reflects that reported in the wider social learning literature (Lebel et al., 2010; Pahl-Wostl, 2009; Fernández-Giménez et al., 2019), with shared assumptions and values emerging as the farmers learnt from and about each other. Facilitation was key to creating an arena for learning and enacting the process design, with the group guided through critical discussion and provided with space for reflection (Pennington et al., 2013; Ensor and Harvey, 2015). Rather than being a prescriptive process, new shared values that AST innovation should satisfy emerged from within the group as members together grew in confidence to express and explore their dissatisfaction with, for example, the direction of technological change, or the risks forced upon them by limited technology choice (c.f. Macmillan and Benton, 2014).

Cognitive learning emerged most clearly during the second and third phases, when a new potential technology was proposed; when the engineer provided feedback to the group; or when priorities or design attributes were agreed. Frames were a variable but ever-present factor in these group discussions. Cycles of issue framing and re-framing – identifying and re-thinking the main issues and boundaries of the problems – underpinned learning in the NE group, enabled by a growing confidence and ease exhibited by group members as they recognised or were challenged by each other's' understandings. Discussions among the group provided an “activating event”, stimulating group members to co-construct new frames in order to make sense of new information (Cranton, 2002, p. 66; see also Pahl-Wostl et al., 2007). In the SB group re-framing was less clear cut: the challenge of wireless signal coverage was presented in a technical frame that remained throughout the co-design process. The overarching issue of farmer safety drew in multiple frames (feelings of safety, technical feasibility, estate responsibility), which were navigated by expanding the co-design process to include the institutional context for decision making on lone worker safety. Expanding the stakeholder group (to include the health and safety officer) provided the trigger towards apparently irreconcilable frames being addressed, as the discussion shifted towards a management system capable of simultaneously satisfying diverse interests. The presence of multiple frames reflected these very different perspectives on lone worker safety; resolution required acknowledgement of alternative frames without the abandonment of any. As Pahl-Wostl et al. (2007 p. 4) note, “When differences can be dealt with constructively by addressing them and trying to connect them instead of avoiding or escalating them, new possibilities can be discovered and social learning becomes possible.”

The centrality of frames highlights the role of prior experience and understandings as the starting point for learning (Kolb, 1984; Novak, 2010), most evident when famers and the engineer each made sense of new information in relation to their current practices and knowledge. Focused around the narrowing of the issues and potential technologies, the back-and-forth flow of these conversations enabled the farmers and engineer to build a deeper appreciation of each other's understandings and practices, strengthening relationships between the group members while also inviting the group to think creatively about what science and technology could and should enable them to do. Thus, while facilitation was principally directed towards the acquisition of new knowledge by the farmers (of technologies) and engineer (of farm context), these discussions were informed by the critical reflections of the first phase, enabling a virtuous cycle of cognitive, normative and relational learning to emerge (Pennington et al., 2013; Fernández-Giménez et al., 2019) without a noticeable trade-off between them (c.f. McFadgen and Huitema, 2017). As noted in other contexts, reframing can lead to the integration of new concepts, but may also extend to the emergence of new priorities, values and ways to interpret situations (Smith et al., 2016). These contextualising discussions supported interpretation of new information and generated new, shared knowledge. In the process, the values expressed within the farmers' and the engineer's past experiences of innovation were challenged (“We've been channelled and focussed to making our equipment, our time do more […] so ultimately we are programmed to run to stand still”). This in turn reinforced the shared narrative of AST that had been developed in the first phase. At the same time, flux in the assumptions about what technology can and should achieve opened space for imagination and creativity (“You could use it for…”; “It will enable me to…”; “‘If it can do…, then…”). This led to the prioritisation of potential technologies and design attributes that better reflected the farmers’ underlying interests and contexts. Thus, by orientating the group engagement process around the space to explore and challenge histories and contexts of AST, and by drawing on social learning principles to facilitate interaction between the different expertise of farmers and between farmers and engineers, learning emerged that interleaved technology co-design with incremental refinement of the shared norms and values embedded in the innovation process itself.

## 6 Conclusion

There is now wide agreement that agricultural science and technology needs to move away from a model that isolates farmers as technology adopters. This paper contributes to a growing literature that documents diverse examples of farmer-led innovation by providing an analytical focus on the forms of learning that take place. As the results and discussion demonstrate, this focus helps deepen understanding of key mechanisms and processes that define and deliver innovation. Cycles of learning emerged from the farmers' critical reflection of their own experience of AST: this focus on the external context disrupted established patterns of thought and practice about innovation for the farmers and engineer, building a shared perception that the goals and values of innovation required reassessment. While undeniably present, the surfacing of underlying epistemological conflicts between stakeholders – the farmers, engineer and wider stakeholders – was secondary to the reassessment of the norms and values of the wider context in terms of precipitating learning. In what followed, cognitive, normative and relational learning were mutually interdependent and equally significant, building iteratively rather than linearly: the farmers and engineer assessed new information and reappraised existing situations; they did so informed by and informing a shift in understanding of their goals for technology; and in so doing they relied on and developed the trust and confidence needed to acknowledge or challenge each other's perspectives. The co-design of new technology was thus inseparable from the emergence of shared values to guide its delivery. While structured by the overall objective of identifying and narrowing issues and projects, the constructive reinforcement between the three forms of learning iteratively redefined both the process and outcome. Overall, this points to the significance of explicitly securing opportunities for cognitive, normative and relational learning within farmer-led innovation, and suggests priorities for process design should focus on modalities that open up spaces that explicitly recognise power and context so as to negotiate both the purpose and products of the innovation process.

Emphasis in this work was placed on building the legitimacy of farmer experiences and knowledge among the engineer and experts that were brought into the process, against the backdrop of contextual shifts in the policy and funding environment towards farmer-led innovation. Not considered was learning that may have taken place beyond the group setting, in wider networks or systems, or the potential for systemic and institutional change. While no single project can explore the mechanisms and processes at play at all temporal, spatial or institutional scales, there would be clear benefits for future action-research to investigate the potential for farmer-led innovation to catalyse wider learning across innovation systems. Such a shift may, for example, entail an analytical focus on learning among organisations and networks, rather than individuals. Equally, future research would benefit from considering the advantages provided by a facilitator that is independent of, rather than embedded in, the research process. Clear and unambiguous roles would provide both researcher and facilitator with more time and flexibility, and offer benefits in terms of detailed participant observation data. Balanced against this are the insights that can be built through a deeper relationship between stakeholders and the researcher, and between the process of facilitation and the underpinning design principles, that comes with the dual researcher-facilitator role. Both of these aspects were important in developing the findings revealed in this paper.

## Declaration of Competing Interest

The authors declare that they have no known competing financial interests or personal relationships that could have appeared to influence the work reported in this paper.

## Footnotes

1 Sumo is a brand of farm machinery that is known for being heavy duty, robust and able to work in harsh conditions. The farmers refer to it here - jokingly - in the sense of ‘if the new technology can survive interaction with the Sumo, it can survive anything on the farm’. As F1 explains: “They're great big bits of metal, Annemarieke, which will smash anything”.
